# CCR7 Controls Thymus Recirculation, but Not Production and Emigration, of Foxp3^+^ T Cells

**DOI:** 10.1016/j.celrep.2016.01.003

**Published:** 2016-01-28

**Authors:** Jennifer E. Cowan, Nicholas I. McCarthy, Graham Anderson

**Affiliations:** 1 MRC Centre for Immune Regulation, Institute for Immunology and Immunotherapy, University of Birmingham, Birmingham B15 2TT, UK

## Abstract

Current models of Foxp3^+^ regulatory T cell (Treg) development involve CCR7-mediated migration of thymocytes into the thymus medulla to enable essential interactions with medullary epithelium. However, increased Foxp3^+^ thymic Treg numbers in *Ccr7*^*−/−*^ mice challenge this view, and the role of CCR7 in Treg development, emigration, and/or recirculation is unknown. Here, we have examined CCR7 and Rag2pGFP levels during Treg development and generated Rag2pGFP*Ccr7*^−/−^ mice to study its impact on the intrathymic Treg pool. We reveal surprising developmental heterogeneity in thymocytes described as Treg precursors, showing that they contain recirculating CCR6^+^CCR7^−^Rag2pGFP^−^ T cells. Although CCR7 defines bona fide Rag2GFP^+^ Treg precursors, it is not required for Treg production and emigration. Rather, we show that lack of CCR7 renders the thymus more receptive to Treg thymus homing. Our study reveals a role for CCR7 in limiting Treg recirculation back to the thymus and enables separation of the mechanisms controlling Treg production and thymic recirculation.

## Highlights

• Chemokine receptors define recirculating and newly selected Tregs
• CCR7 is dispensable for Treg development and thymus egress
• CCR7 is required to rate-limit the thymus recirculation of peripheral CCR6^+^ Tregs

## Graphical Abstract

**Figure:**
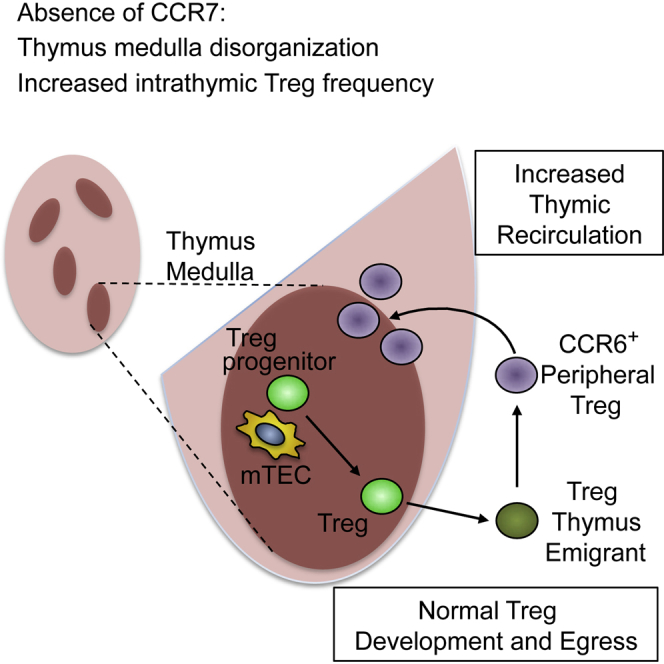

## Teaser

Current models of Foxp3^+^ Treg development indicate that CCR7 controls thymus medulla entry for Treg precursor generation. By redefining newly produced Treg precursors, Cowan et al. show that, while CCR7 is dispensable for Treg development, it influences the intrathymic Treg pool by rate-limiting peripheral Treg recirculation back to the thymus.

## Introduction

In the thymus, positive selection generates subsets of CD4^+^ αβT cells that are functionally distinct, and immature CD4^+^CD8^+^ thymocytes represent the precursors of both CD4^+^ conventional helper and Foxp3^+^ regulatory T cells (Tregs). While positive selection of conventional CD4^+^ thymocytes requires cortical thymic epithelial cells (cTEC) (Murata et al., 2007), their further differentiation can take place independently of the thymic medulla, which instead operates as a site for negative selection (Boehm et al., 2003, Cowan et al., 2013, Koble and Kyewski, 2009). In contrast, medullary thymic microenvironments provide essential support for the differentiation of Foxp3^+^ T cells, (Coquet et al., 2013, Cowan et al., 2013), including the generation of CD25^+^Foxp3^−^ and CD25^−^Foxp3^+^ precursors (Lio and Hsieh, 2008, Tai et al., 2013). Although pathways in the development of medullary thymic epithelial cells (mTEC) are becoming clearer (Ohigashi et al., 2015, Takaba et al., 2015), the role of the medulla in Treg generation is still poorly understood. Nevertheless, entry into the medulla is known to be triggered by positive selection, with induction of CCR7 expression altering the migratory ability of newly selected thymocytes and enabling access medullary thymic microenvironments (Kwan and Killeen, 2004, Liston et al., 2008, Ueno et al., 2004). In *Ccr7*^−/−^ mice, the ability of conventional thymocytes to enter the medulla is dramatically altered, fitting well with their reported breakdown in negative selection (Davalos-Misslitz et al., 2007, Kurobe et al., 2006, Nitta et al., 2009). Paradoxically however, increased Foxp3^+^ Treg numbers in the thymus of *Ccr7*^*−/−*^ mice (Cowan et al., 2014, Schneider et al., 2007) are at odds with current models of Foxp3^+^ T cell development, in which CCR7 controls access to the medullary stromal cells that support the generation and maturation of Treg precursors.

Direct measurement of the frequency and requirements of Foxp3^+^ T cell development is made difficult by the developmental heterogeneity of intrathymic Foxp3^+^ T cells, which consist of both newly generated cells and mature cells that re-enter the thymus from the periphery (Hale and Fink, 2009). Significantly, analysis of Rag2pGFP transgenic mice indicates that thymus-recirculating Tregs constitute approximately 30%–50% of the total thymic Treg pool in young adult mice (McCarthy et al., 2015, McCaughtry et al., 2007, Thiault et al., 2015, Yang et al., 2014). Consequently, the mechanisms controlling intrathymic Foxp3^+^ T cell development remain poorly understood. In order to allow separate analysis of de novo Treg development and thymic recirculation, we have mapped CCR7 expression to distinct maturation stages revealed by Rag2pGFP levels and generated Rag2pGFP*Ccr7*^*−/−*^ mice to directly examine the impact of CCR7 on the intrathymic Treg pool. Importantly, we show that intrathymic CD25^+^Foxp3^−^ and CD25^−^Foxp3^+^ populations of CD4^+^ thymocytes, previously reported as Treg precursors, contain mature recirculating Rag2pGFP^−^ T cells that lack CCR7 but express CCR6, a marker of effector Tregs. In contrast, and consistent with a role for CCR7 during Treg development, newly generated Rag2pGFP^+^ Treg precursors and their Treg progeny are CCR6^−^CCR7^+^. However, despite this expression pattern, de novo Rag2pGFP^+^ Tregs are generated and exported normally in the absence of CCR7. Moreover, using both Rag2pGFP levels and thymus transplantation to assess Treg recirculation, we show that increased Treg numbers in *Ccr7*^*−/−*^ mice occur as a result of increased thymic Treg homing. Collectively, our study identifies bona fide Treg precursors, allowing accurate analysis of their frequency and developmental requirements, and reveals a role for CCR7 in limiting the contribution of thymus-recirculating cells to the intrathymic Treg pool.

## Results and Discussion

### CD25^+^Foxp3^−^ and CD25^−^Foxp3^+^ Treg Precursor Populations Contain Thymus-Recirculating Mature T Cells

**Figure 1:**
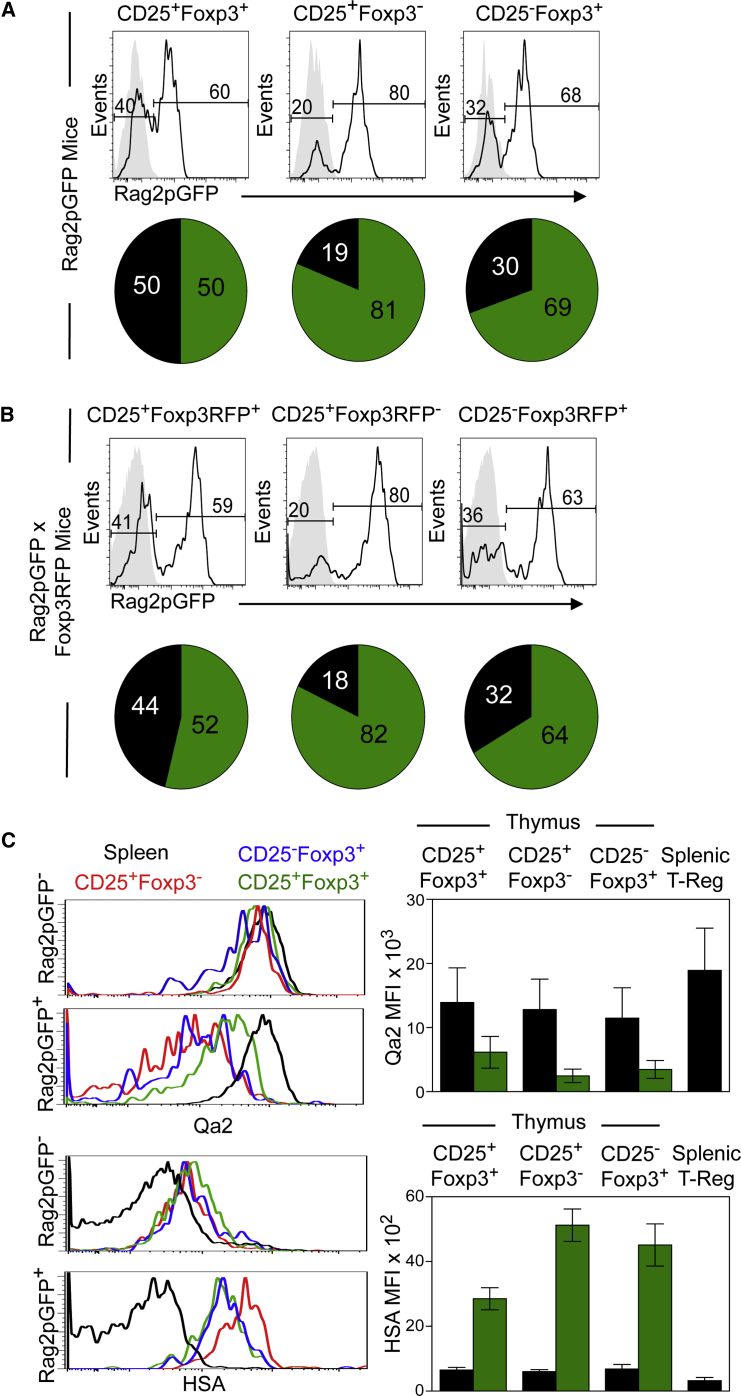
Redefining Stages during Intrathymic Development of Foxp3^+^ Treg (A) CD25^+^Foxp3^+^, CD25^+^Foxp3^−^, and CD25^−^Foxp3^+^ subsets of TCRβ^hi^CD4^+^ thymocytes from Rag2pGFP mice were analyzed for expression of GFP (black lines). Gray histograms show fluorescence levels of WT cells. Pie charts show the percentage medians of Rag2pGFP^−^ (black) and Rag2pGFP^+^ (green) cells in the histograms. The SDs of populations in the pie charts are as follows: CD25^+^Foxp3^+^GFP^+^, 12.8; CD25^+^Foxp3^+^GFP^−^, 12.8; CD25^+^Foxp3^−^GFP^+^, 4.8; CD25^+^Foxp3^−^GFP^−^, 4.8; CD25^−^Foxp3^+^GFP^+^, 8.5; and CD25^−^Foxp3^+^GFP^−^, 8.5. Data represent at least three separate experiments. (B) Levels of Rag2pGFP and Foxp3RFP in CD25/Foxp3 subsets of TCRβ^hi^SP4^+^ thymocytes from Rag2pGFP × Foxp3RFP dual reporter mice. Gray histograms indicate control fluorescence levels in Foxp3RFP single reporter adult mice. Pie charts show the percentage medians of Rag2pGFP^−^ (black) and Rag2pGFP^+^ (green) cells, and the SDs of Rag2pGFP × Foxp3RFP populations are as follows: CD25^+^Foxp3^+^GFP^+^, 11.9; CD25^+^Foxp3^+^GFP^−^, 10; CD25^+^Foxp3^−^GFP^+^, 8; CD25^+^Foxp3^−^GFP^−^, 8; CD25^−^Foxp3^+^GFP^+^, 16; CD25^−^Foxp3^+^GFP^−^, 14. Data represent at least three separate experiments. (C) Thymic subsets of CD25/Foxp3 TCRβ^hi^CD4^+^ cells were divided into Rag2pGFP^−^ and Rag2pGFP^+^ cells, and levels of Qa2 and HSA are shown for each. The black lines in all histograms represent Rag2pGFP^−^ Tregs from spleen. The bar charts show Qa2/HSA mean fluorescence intensity (MFI) of the indicated thymic Rag2pGFP^+^ (green bars) and Rag2pGFP^−^ (black bars) subsets, with the MFI of total Tregs from spleen shown for comparison. Error bars represent SEM.

Intrathymic Foxp3^+^ Treg generation is a multi-stage process, and distinct subsets of CD4^+^ thymocytes with CD25^+^Foxp3^−^ and CD25^−^Foxp3^+^ phenotypes have been reported as Treg precursors (Lio and Hsieh, 2008, Tai et al., 2013). To better define Treg precursors, and to analyze their development and frequency in relation to their mature CD25^+^Foxp3^+^ Treg progeny, we analyzed these populations in Rag2pGFP mice, where levels of GFP expression are an indicator of maturational status (Boursalian et al., 2004). Thymocytes from adult Rag2pGFP mice were analyzed by flow cytometry for their expression of a panel of differentiation markers, including Foxp3. As shown previously (McCaughtry et al., 2007), we found the intrathymic CD25^+^Foxp3^+^ Treg pool to be developmentally heterogeneous, containing both newly produced Rag2pGFP^+^ cells and recirculating Rag2pGFP^−^ Tregs (Figure 1A). Surprisingly, we also detected heterogeneity in the CD25^+^Foxp3^−^ and CD25^−^Foxp3^+^ subsets of CD4^+^ thymocytes previously described as precursors of mature CD25^+^Foxp3^+^ Tregs. Approximately 20% of CD25^+^Foxp3^−^ and 30% of CD25^−^Foxp3^+^ cells within TCRβ^hi^CD4^+^ thymocytes were Rag2pGFP^−^ (Figure 1A), with fluorescence levels comparable to that seen in the complementary populations of thymocytes from wild-type (WT) mice (Figure 1A, gray histograms). To exclude the possibility that the presence of cells lacking detectable GFP in putative Treg precursor populations was due to the loss of GFP protein caused by permeabilization methods used with anti-Foxp3 antibodies, we crossed Rag2pGFP mice with Foxp3RFP (red fluorescent protein) reporter mice. Thymocyte analysis of adult Rag2pGFP/Foxp3RFP dual reporter mice (Figure 1B) showed that approximately 20% of CD25^+^Foxp3RFP^−^ and 30% of CD25^−^Foxp3RFP^+^ subsets of CD4^+^ thymocytes contained Rag2pGFP^−^ cells (Figure 1B), again with levels of fluorescence comparable to that of complementary populations from Foxp3RFP reporter mice (Figure 1B, gray histograms).

The aforementioned findings are consistent with the idea that, as with CD25^+^Foxp3^+^ cells, both CD25^+^Foxp3^−^ and CD25^−^Foxp3^+^ cells contain mature T cells that have re-entered the thymus from the periphery. To examine this possibility further, we compared the phenotype of Rag2pGFP^−^ cells in the thymus with that of Rag2pGFP^−^ Tregs from the spleen. Thymic Rag2pGFP^−^CD25^+^Foxp3^−^ and Rag2pGFP^−^CD25^−^Foxp3^+^ cells expressed high levels of Qa2 and low levels of heat-stable antigen (HSA), similar to splenic Rag2GFP^−^ Tregs (Figure 1C). In contrast, and consistent with their progenitor status, Rag2pGFP^+^CD25^+^Foxp3^−^ and Rag2pGFP^+^CD25^−^Foxp3^+^ cells were Qa2^low^HSA^hi^ (Figure 1C). Thus, analysis of single Rag2pGFP and dual Rag2pGFP/Foxp3RFP reporter mice reveals unexpected developmental heterogeneity within intrathymic cells initially defined as Treg precursors. Specifically, we identified a substantial proportion of thymic CD25^+^Foxp3^−^ and CD25^−^Foxp3^+^ cells as mature recirculating T cells, a finding that enables accurate analysis of the frequency, phenotype, and developmental requirements of de novo Treg precursors.

### CCR7 and CCR6 Distinguish Developing Tregs from Recirculating T Cells

**Figure 2:**
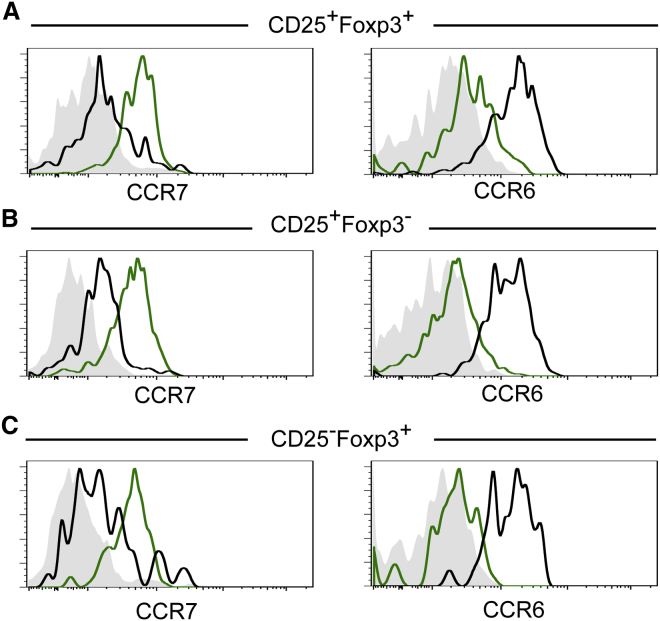
Differential Expression of CCR7 and CCR6 Distinguishes Newly Produced and Recirculating Tregs in the Thymus (A–C) Thymocytes from adult Rag2pGFP mice were stained for expression of CD4, CD8, TCRβ, CD25, Foxp3, CCR7, and CCR6. Rag2pGFP^−^ (black) and Rag2pGFP^+^ (green) subsets of TCRβ^hi^CD4^+^CD25^+^Foxp3^+^ (A), TCRβ^hi^CD4^+^CD25^+^Foxp3^−^ (B), and TCRβ^hi^CD4^+^CD25^−^Foxp3^+^ (C) thymocytes were then analyzed for levels of CCR7 and CCR6. Gray histograms in the left panels show fluorescence levels in cells from *Ccr7*^*−/−*^ mice stained with anti-CCR7, while gray histograms in the right panels show WT cells stained with an isotype control for anti-CCR6. Data are representative of at least three experimental repeats.

Given the discrepancy between current models of intrathymic Foxp3^+^ T cell development and the expansion of Foxp3^+^ Tregs in the thymus of *Ccr7*^*−/−*^ mice (Cowan et al., 2014, Schneider et al., 2007), we analyzed CCR7 and Rag2pGFP expression in CD4 thymocyte subsets defined by CD25 and Foxp3. Interestingly, while CCR7 was uniformly expressed by the Rag2pGFP^+^ subsets of CD25^+^Foxp3^+^, CD25^+^Foxp3^−^, and CD25^−^Foxp3^+^ thymocytes, their Rag2pGFP^−^ counterparts were CCR7^−^ (Figures 2A–2C). Such observations are important, as they explain the previously reported heterogeneity in CCR7 expression by Treg precursor populations (Cowan et al., 2014) that did not take into account the presence of Rag2pGFP^−^ mature T cells. To enable further discrimination of de novo Rag2pGFP^+^ Treg development from recirculating Rag2pGFP^−^ T cells, we screened for expression of other chemokine receptors. Interestingly, CCR6—a chemokine receptor expressed by effector/memory, but not naive, T cells (Campbell, 2015)—is uniformly expressed by Rag2pGFP^−^ cells, but not Rag2pGFP^+^ cells, within all thymic CD25^+^Foxp3^+^, CD25^+^Foxp3^−^, and CD25^−^Foxp3^+^ subsets (Figure 2). Collectively, these findings show that differential CCR6/CCR7 expression separates stages in de novo Treg development from thymus-recirculating cells. Moreover, they also demonstrate the potential for CCR7 to influence the earliest stages of Foxp3^+^ Treg development by enabling the interactions of their precursors with thymic medullary areas.

### CCR7 Is Dispensable for the Thymic Development and Emigration of Foxp3^+^ Tregs

**Figure 3:**
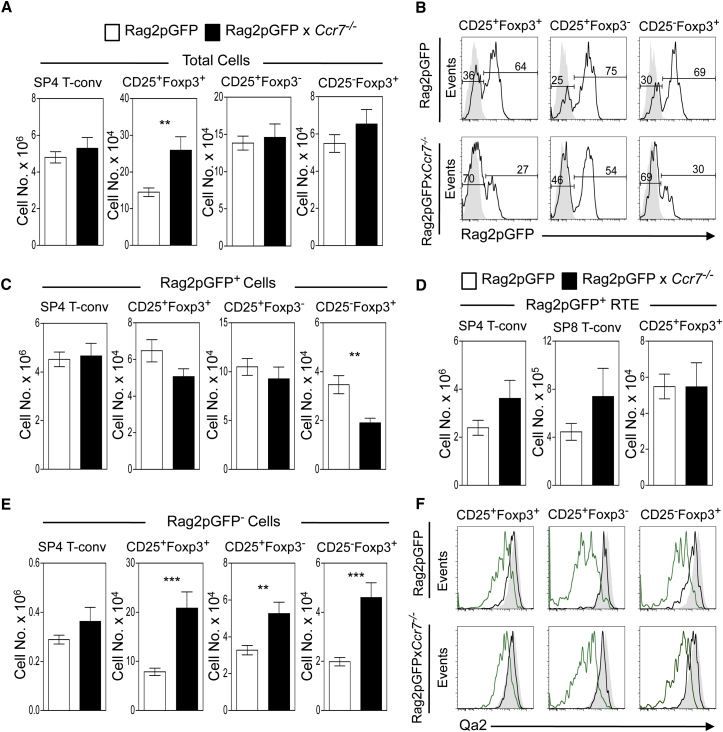
Production and Emigration of Foxp3^+^ Tregs Occurs Independently of CCR7 (A) Comparative analysis of Foxp3^+^ T cell development in Rag2pGFP (white bars) and Rag2pGFP × *Ccr7*^*−/−*^ (black bars) mice. (B) Rag2pGFP levels in indicated subsets of TCRβ^hi^CD4^+^ thymocytes from Rag2pGFP (upper panels) and Rag2pGFP × *Ccr7*^*−/−*^ (lower panels) mice. Gray histograms represent fluorescence levels of WT adult cells. (C) Numbers of Rag2pGFP^+^ cells of the indicated thymocyte subsets from Rag2pGFP (white bars) and Rag2GFP × *Ccr7*^*−/−*^ mice (black bars). (D) Number of CD4 T-conv, CD8, and Treg Rag2pGFP^+^ RTE in Rag2pGFP (white bars) and Rag2pGFP × *Ccr7*^*−/−*^ mice (black bars). (E) Frequency of Rag2pGFP^−^ cells in the indicated TCRβ^hi^CD4^+^ thymocyte subsets from Rag2pGFP (white bars) and Rag2pGFP × *Ccr7*^*−/−*^ (black bars) mice. (F) Qa2 levels in Rag2pGFP^+^ (green) and Rag2pGFP^−^ (black) thymocytes from Rag2pGFP (upper histograms) and Rag2pGFP × *Ccr7*^*−/−*^ mice (lower histograms). Shaded histograms show Qa2 levels of splenic Rag2pGFP^-^ Foxp3^+^ Treg cells for comparison. Error bars display SEM, and all graphs are representative of at least eight mice per group and three experimental repeats. In an unpaired Student’s t test, ^∗∗^p < 0.01; ^∗∗∗^p < 0.001.

Given the pattern of CCR7 expression described earlier, we next investigated its role in the development, egress, and recirculation of the thymic Foxp3^+^ T cell pool. We crossed *Ccr7*^*−/−*^ mice to Rag2pGFP mice and used GFP expression to allow for the separate analysis of de novo Foxp3^+^ Treg generation and thymus recirculation. Consistent with previous reports on *Ccr7*^*−/−*^ mice, total CD25^+^Foxp3^+^ Tregs were increased in the thymus of Rag2pGFP*Ccr7*^−/−^ mice (Figure 3A). Interestingly, when we examined Rag2pGFP expression in all CD25/Foxp3 subsets, in the absence of CCR7 we saw a decrease in the proportion of Rag2pGFP^+^ cells and a concomitant increase in Rag2pGFP^−^ cells (Figure 3B). However, while the number of Rag2pGFP^+^CD25^−^Foxp3^+^ cells was also reduced, numbers of Rag2pGFP^+^CD25^+^Foxp3^−^ Treg precursors and newly generated Rag2pGFP^+^CD25^+^Foxp3^+^ Tregs in WT and *Ccr7*^*−/−*^ mice did not change (Figure 3C). Moreover, no decrease in Rag2pGFP^+^CD25^+^Foxp3^+^ recent thymus emigrants (RTE) was observed in *Ccr7*^*−/−*^ mice (Figure 3D). Collectively, these findings show that, despite its key role in cortex-to-medulla migration and its expression during de novo Foxp3^+^ T cell development, CCR7 is not required for the effective intrathymic generation of Foxp3^+^ Tregs or for their emigration from the adult thymus. Importantly, these findings fit well with a previous report showing that CCR7-deficient Tregs possess in vitro suppressive activity comparable to that of WT cells (Schneider et al., 2007), strengthening the notion that the effective generation of Foxp3^+^ Tregs, including their acquisition of functional competence, can occur independently of CCR7 expression.

### CCR7 Limits the Contribution of Recirculating Tregs to the Intrathymic Pool

We saw, in striking contrast to Rag2GFP^+^ cells, an increase in Rag2pGFP^−^ cells within CD25^+^Foxp3^+^ and CD25^−^Foxp3^+^/CD25^+^Foxp3^−^ populations in *Ccr7*^*−/−*^ mice (Figure 3E), suggesting that, in the absence of CCR7, expansion of the intrathymic Treg pool is caused by increased thymus recirculation of mature peripheral T cells. Consistent with this, Rag2pGFP^−^ cells in the thymus of both WT Rag2pGFP and Rag2pGFPx*Ccr7*^*−/−*^ mice had levels of Qa2 comparable to that in peripheral Tregs (Figure 3F) and expressed CCR6 (data not shown), a phenotype of thymus-recirculating cells (Figure 2).

**Figure 4:**
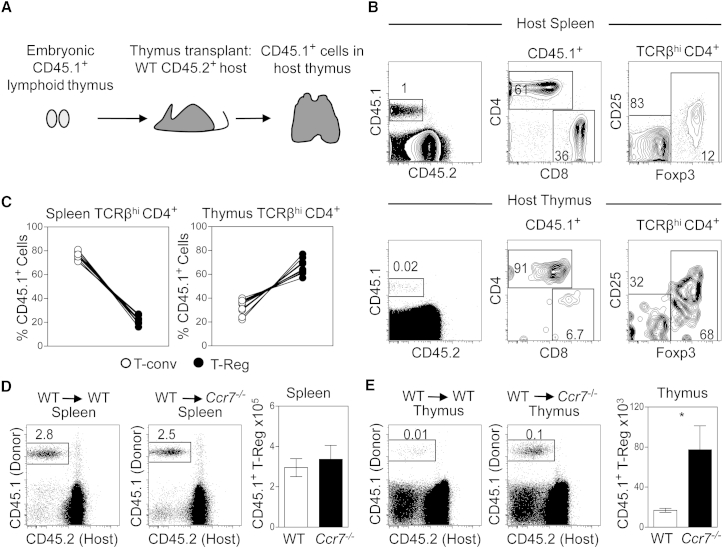
CCR7 Limits the Contribution of Recirculating Tregs to the Intrathymic Pool (A) Diagrammatic representation of the kidney capsule thymus-grafting system, where CD45.1^+^ WT thymus lobes were transplanted under the kidney capsule of either WT or *Ccr7*^*−/−*^ CD45.2^+^ host mice, and the presence of graft-derived CD45.1^+^CD45.2^−^ cells was analyzed in the recipient thymus and spleen. (B) Fluorescence-activated cell sorting (FACS) analysis of spleen (upper panels) and host thymus (lower panels) cells identifying CD45.1^+^ graft-derived cells, which were further analyzed for expression of TCRβ, CD4, CD8, CD25, and Foxp3 as indicated. (C) Graphs represent the percentage of CD45.1^+^TCRβ^hi^CD4^+^Foxp3^−^ T-conv (white circles) and CD45.1^+^ TCRβ^hi^CD4^+^Foxp3^+^ Tregs (black circles) in the host spleen (left graph) and thymus (right graph). (D and E) Frequency of WT CD45.1^+^ donor cells in the host spleen (D) or thymus (E) of either WT or *Ccr7*^*−/−*^ CD45.2^+^ host mice. Bar graphs show the number of donor-derived CD45.1^+^CD25^+^Foxp3^+^ Tregs in the thymus and spleen of WT (white bars) or *Ccr7*^*−/−*^ (black bars) hosts. Error bars show SEM in all bar charts and are representative of at least six mice per condition and three experimental repeats. In an unpaired Student’s t test, ^∗^p < 0.05.

Although the phenotypic data described earlier supports the idea of increased thymus homing in the absence of CCR7 expression, we adopted a thymus transplantation model to directly examine thymus recirculation (Figure 4A). In initial experiments, we transplanted embryonic WT lymphoid thymus lobes from CD45.1^+^ donors under the kidney capsule of CD45.2^+^ WT hosts. In this system, a single cohort of donor-derived T cell precursors resident in the graft at the time of transplant generates a wave of mature T cells that can be tracked within host tissues. After 4–6 weeks, we assessed host thymus and spleen for the presence of CD45.1^+^CD45.2^−^ graft-derived cells. In spleen (Figures 4B and 4C), we found that the majority of graft-derived cells were Foxp3^−^CD4^+^ and CD8^+^ conventional T cells (T-conv), while approximately 15%–20% of CD45.1^+^CD4^+^ cells were Foxp3^+^ Tregs. Interestingly, the makeup of graft-derived T cells that had migrated into the host thymus was strikingly different, and the majority (approximately 60%–80%) of cells were CD4^+^Foxp3^+^ Tregs (Figures 4B and 4C). Collectively, these findings demonstrate the robustness of this thymus transplantation approach to directly identify and quantitative thymus-homing Tregs. Moreover, they also suggest that, when compared to T-conv generated within the same thymic transplant, Foxp3^+^ Tregs show a preferential capacity for thymus homing.

To directly assess the role of CCR7 in the thymus homing of peripheral Tregs, we next transplanted WT CD45.1^+^ fetal thymus lobes into either CD45.2^+^ WT or CD45.2^+^ *Ccr7*^*−/−*^ hosts. Following transplantation, the presence and frequency of graft-derived CD45.1^+^ Tregs in the thymus and spleen of WT and host *Ccr7*^*−/−*^ mice were determined. Figure 4D shows that the proportion of CD45.1^+^ WT cells in the spleen of WT and *Ccr7*^*−/−*^ hosts was comparable, as was the number of graft-derived Tregs. In contrast, we saw an increase in the proportion of CD45.1^+^ graft-derived cells in the thymus of *Ccr7*^*−/−*^ mice, as compared to WT mice, that correlated with a significant increase in the absolute number of graft-derived CD45.1^+^ Tregs in the *Ccr7*^*−/−*^ thymus (Figure 4E). Collectively, such observations show that the absence of CCR7 results in an increase in the entry of peripheral Tregs back to the thymus, suggesting a role for CCR7 in limiting thymic re-entry. Moreover, they also provide an explanation for the increased size of the intrathymic Treg pool in *Ccr7*^*−/−*^ mice.

Our work is important in understanding intrathymic Foxp3^+^ T cell development and its control. Since the identification of two distinct CD25^+^Foxp3^−^ and CD25^−^Foxp3^+^ precursor pools (Lio and Hsieh, 2008, Tai et al., 2013), their frequency and developmental requirements have been well studied (Hauri-Hohl et al., 2014, Liu et al., 2014, McCarthy et al., 2015). Importantly, we show here that such populations are not uniformly thymocyte precursors and that they, instead, contain mature T cells that have re-entered the thymus from the periphery. Thus, failing to exclude thymic-recirculating cells means that previous studies have likely overestimated the frequency of Treg precursors, which warrants a rethinking of the developmental requirements of Foxp3^+^ T cell development. Relevant to this, redefining de novo Treg precursors as CCR6^−^CCR7^+^ cells should aid in their accurate identification and further study. Additionally, our work argues against current models in which access to the medullary areas that control Treg development requires CCR7-mediated thymocyte migration. Rather, we propose that additional chemokine receptors expressed during initial stages of Foxp3^+^ T cell development ensure that essential interactions with mTEC take place. While patterns of CCR4 expression are suggestive of a role in this process, Treg development is not reduced in *Ccr4*^*−/−*^ mice, even when combined with the absence of CCR7 (Cowan et al., 2014). Further work is required to map the expression, and test the requirement, for defined chemokine receptors during Treg precursor generation. Interestingly, our finding that Foxp3^+^ T cell development and thymus emigration in adult mice occurs in the absence of CCR7 is mirrored by studies on conventional CD4^+^ thymocytes (Ueno et al., 2004). Thus, although Tregs and conventional thymocytes differ in their requirements for the thymus medulla (Cowan et al., 2013), they share an ability to develop and emigrate independently of CCR7. A comparison of the chemokine receptor profiles of CD4^+^ conventional and Treg RTE may aid in the identification of important regulators of thymic emigration.

Finally, our experiments provide insight into the control and significance of thymus-homing T cells. For example, while we show that CCR7 is required to limit re-entry to the thymus, recirculating T cells are CCR6^+^CCR7^−^. Given this lack of CCR7 expression by thymus-homing Tregs, it is unlikely that increased thymic re-entry in *Ccr7*^−/−^ mice is due to a loss of their CCR7-mediated migration. Rather, CCR7 appears to be required indirectly, perhaps during intrathymic T cell development, to limit thymic recirculation. For example, CCR7 expression during thymic selection may enable newly generated Tregs to compete more effectively with recirculating cells for medullary niches. As an additional scenario, the disrupted thymic organization in *Ccr7*^*−/−*^ mice caused by alterations in thymocyte migration (Ueno et al., 2002, Ueno et al., 2004) may also alter the availability of intrathymic niches for Foxp3^+^ Tregs and enable more peripheral T cells to re-enter the thymus. Further work is required to examine the intrathymic positioning of recirculating versus newly generated Tregs in the thymus of *Ccr7*^*−/−*^ mice. In addition, our work is relevant to the possible functional importance of thymus-homing Tregs, which were recently proposed to limit intrathymic Treg development by competing for interleukin-2 (IL-2) availability (Thiault et al., 2015, Weist et al., 2015). However, it is significant that, despite enhanced Treg recirculation in *Ccr7*^*−/−*^ mice, numbers of newly produced CD25^+^Foxp3^+^ Tregs are not changed. Indeed, the only effect on Treg development seen in *Ccr7*^*−/−*^ mice is a reduction in CD25^−^Foxp3^+^Rag2pGFP^+^ precursors, and their lack of CD25 expression suggests that this decrease is unlikely to be caused by competition for IL-2. Thus, Treg recirculation may be limited in its ability to influence de novo Foxp3^+^ T cell development. Further examination of the mechanisms that regulate Treg thymus homing will provide an opportunity to better understand its impact on intrathymic T cell development.

## Experimental Procedures

### Mice

Adult WT, Rag2pGFP transgenic (Yu et al., 1999), and *Ccr7*^*−/−*^ mice (Pahuja et al., 2006) on a CD45.2 C57BL/6 background were used at 6–10 weeks of age. Foxp3RFP/Rag2pGFP dual reporter mice were generated by crossing Rag2pGFP mice to Foxp3RFP mice (Wan and Flavell, 2005). Timed matings of CD45.1^+^ congenic mice were used to generate embryonic day (E)17–19 thymus lobes for transplantation. All mice were housed at the University of Birmingham Biomedical Services Unit in accordance with local and national Home Office guidelines.

### Antibodies and Flow Cytometry

The following antibodies were used for flow cytometric analysis: Brilliant Violet (BV) 711 anti-CD4 (clone GK1.5; BioLegend), BV785 anti-CD8 (clone 53-6.7; BioLegend), Allophyocyanin eFluor 780 anti-TCRβ (clone H57-597; eBioscience), Biotinylated anti-Qa2 (clone 695H1-9.9; BioLegend), PECy7 anti-CD45.1 (clone A20; eBioscience), Allophyocyanin/A700 anti-CD25 (clone PC61.5; eBioscience), BV605 anti-CCR6 (clone G034E3; BioLegend), Allophyocyanin anti-CD45.2 (clone 104; eBioscience), PE anti-CCR7 (clone 4B12: eBioscience), PE/eFluor 450 anti-Foxp3 (clone FJK-16 s; eBioscience), and PE anti-HSA/CD24 (clone M1/69; eBioscience). Biotinylated antibodies were detected with PECy7-conjugated streptavidin (eBioscience). For the intracellular staining of Foxp3, cells were fixed and permeabilized using either the Foxp3/Transcription Factor Staining Buffer Set (eBioscience) or the BD Cytofix/Cytoperm Kit (BD Biosciences) to preserve the GFP signal. Both kits were used according to the manufacturer’s protocol. Flow cytometric data were acquired using a BD LSRFortessa and analyzed using FloJo software (Cowan et al., 2013).

### Analysis of RTE

Frequency of RTE was quantitated by analysis of Rag2pGFP^+^ T cells in the peripheral lymphoid organs of WT and *Ccr7*^*−/−*^ mice. As the distribution of T cells in these tissues of WT and *Ccr7*^*−/−*^ mice is unequal, we used a previously reported approach (Berzins et al., 1998, Ueno et al., 2004) to calculate the number of RTE using the formula: number of CD4^+^ RTE = number of Rag2pGFP^+^CD4^+^ T cells in the spleen + 2 × (total number of Rag2pGFP^+^CD4^+^ T cells in pooled mesenteric, inguinal, and axillary lymph nodes).

### Thymus Transplantation

Freshly isolated CD45.1^+^ congenic lymphoid E17–19 thymus lobes were grafted under the kidney capsule of either WT or *Ccr7*^*−/−*^ adult mice (Cowan et al., 2013). At 4–6 weeks after transplantation, the frequency of graft-derived CD45.1^+^Foxp3^+^ T cells in the host thymus and spleen was determined by flow cytometry.

## Author Contributions

G.A. and J.E.C. conceived the study and designed all the experiments. J.E.C. and N.I.M. performed all of the experiments and analyzed data. G.A. and J.E.C. wrote the paper.
